# Mutation of key lysine residues in the Insert B region of the yeast dynamin Vps1 disrupts lipid binding and causes defects in endocytosis

**DOI:** 10.1371/journal.pone.0215102

**Published:** 2019-04-22

**Authors:** Iwona I. Smaczynska-de Rooij, Christopher J. Marklew, Sarah E. Palmer, Ellen G. Allwood, Kathryn R. Ayscough

**Affiliations:** Department of Biomedical Science, University of Sheffield, Sheffield, United Kingdom; Institut Curie, FRANCE

## Abstract

The yeast dynamin-like protein Vps1 has roles at multiple stages of membrane trafficking including Golgi to vacuole transport, endosomal recycling, endocytosis and in peroxisomal fission. While the majority of the Vps1 amino acid sequence shows a high level of identity with the classical mammalian dynamins, it does not contain a pleckstrin homology domain (PH domain). The Dyn1 PH domain has been shown to bind to lipids with a preference for PI(4,5)P_2_ and it is considered central to the function of Dyn1 in endocytosis. The lack of a PH domain in Vps1 has raised questions as to whether the protein can function directly in membrane fusion or fission events. Here we demonstrate that the region Insert B, located in a position equivalent to the dynamin PH domain, is able to bind directly to lipids and that mutation of three lysine residues reduces its capacity to interact with lipids, and in particular with PI(4,5)P_2_. The Vps1 KKK-AAA mutant shows more diffuse staining but does still show some localization to compartments adjacent to vacuoles and to endocytic sites suggesting that other factors are also involved in its recruitment. This mutant selectively blocks endocytosis, but is functional in other processes tested. While mutant Vps1 can localise to endocytic sites, the mutation results in a significant increase in the lifetime of the endocytic reporter Sla2 and a high proportion of defective scission events. Together our data indicate that the lipid binding capacity of the Insert B region of Vps1 contributes to the ability of the protein to associate with membranes and that its capacity to interact with PI(4,5)P_2_ is important in facilitating endocytic scission.

## Introduction

Dynamins are a conserved family of proteins involved in membrane fusion and fission[1–3]. While mammalian dynamins are known to be involved in several membrane trafficking events, the role of dynamin-1 in endocytosis is the best characterised role of this protein family. Dynamin-1 has been proposed to act largely in a mechanical way to encircle the neck of invaginated endocytic vesicles facilitating the scission of vesicles during endocytosis [4–6].

The ability of classical dynamins to associate with membranes has been largely attributed to their PH domains. On recruitment to the plasma membrane, the PH domain can bind to PI(4,5)P_2_ and insert peptide loops into the outer leaflet of the bilayer. The combined function of the PH domain loop insertion and the assembly of dynamin into higher order ring and helical structures is proposed to drive the formation of hemi-fission intermediates, which, on hydrolysis of GTP, will drive membrane scission and release of endocytic vesicles[7, 8]. Structural analyses have led to the suggestion that the PH domain is able to undergo a major conformation shift relative to the rest of the dynamin protein. In the ‘closed’ state, found in crystal structures the PH domains would not be able to bind liposomes and would be likely to obstruct the formation of higher order dynamin structures [9, 10]. In contrast, cryo-EM studies of dynamin bound to lipid templates indicate that the PH domain is located at the ‘base’ of the higher order structures where it interacts with the membrane [11]. Recent studies have used FRET to detect this large conformational change in the PH domain relative to the rest of the dynamin molecule. These studies suggest that this flexibility underpins both membrane binding and oligomerization [12].

In terms of lipid binding specificity, the most intensively studied dynamin, Dyn1, has been shown to bind PI(4,5)P_2_ through its PH domain [13, 14]. However, the lipid-binding properties of the PH domain are not required for dynamin targeting to clathrin-coated endocytic pits suggesting its localization is more likely mediated through protein-protein interactions [8, 15].

Dynamin-related proteins have a domain structure highly related to that of the classical dynamins, containing both GTPase (head) and middle domains but they lack PH domains. Instead, at an equivalent position, they have a region referred to as Insert B. This region is variable between proteins and is generally considered to be of low sequence complexity. The lack of a PH domain has led to the suggestion that the function of these proteins in catalyzing membrane fusion and fission is attributable to their association with membrane binding partners [16]. There is however evidence for direct interactions between different Insert Bs and specific membrane lipids, and also that these interactions are important for the full functionality of the protein in membrane fission. For example, dynamin-related protein 1 (Drp1) has been shown to interact with cardiolipin-enriched membranes (typical of mitochondria) via a 4 lysine module located within its Insert B region [17]. In addition, the human interferon-induced large GTPase MxA protein, which is also a member of the dynamin superfamily, has been shown to interact with lipids via its unstructured L4 loop that lies in an equivalent position to the PH and Insert B regions [18]. MxA lipid binding has also been shown to be attributable to a stretch of 4 lysine residues. These findings have supported the idea that the Insert B region in dynamin-related proteins, similar to the dynamin PH domain, may mediate key interactions with membranes.

Vps1 is a dynamin-like protein, and in yeast is the only dynamin with clear functions in membrane trafficking pathways [19–23]. The two other dynamin-like proteins in *S*. *cerevisiae*, Dnm1 and Mgm1 both function in mitochondrial fission/fusion [24, 25], with Dnm1 having an additional function in peroxisomal fission [26–28]. Like other dynamins, Vps1 is predicted to have a GTPase domain and a middle region. However, in common with non-classical dynamins it does not have a PH domain but the alternative Insert B. Despite the absence of a PH domain, Vps1 can localize to both endosomes and endocytic sites, and is able to directly bind to, and tubulate, liposomes [20, 23, 29]. Real time analysis of single endocytic events demonstrates that deletion of Vps1 compromises scission such that retraction back to the plasma membrane is commonly observed [20]. This phenotype suggests that Vps1 is functioning in a similar way to Dyn1 or Dyn2 in providing a key facet of the fission machinery during endocytosis.

In this study we sought to clarify the importance of the Vps1 Insert B region and its possible role in lipid binding. We have also generated a mutation with reduced lipid binding capacity and report the effect of this mutation on different functions of Vps1 throughout the endo-lysosomal system.

## Results

### Motifs within Vps1 Insert B show high levels of conservation in fungal species

**Fig 1:**
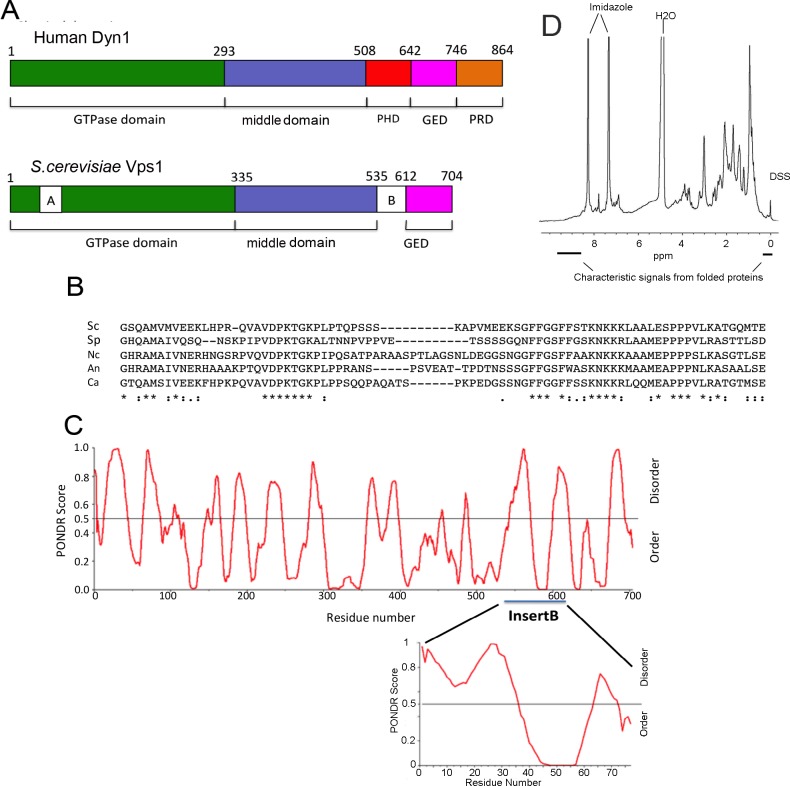
Analysis of Insert B in Vps1. (A) Schematic diagram showing the domain organisation of classical human dynamin Dyn1 and *S*. *cerevisiae* Vps1, including Insert A in the GTPase domain and Insert B immediately prior to the GED domain. (B) Alignment of Insert B from Vps1 and other close fungal Vps1 homologues. Sc *Saccharomyces cerevisiae*, Sp *Schizosaccharomyces pombe*, Nc *Neurospora crassa*, An *Aspergillus nidulans*, and Ca *Candida albicans*. (C) PONDR analysis of Vps1. Prediction of Naturally Disordered Regions (PONDR) score of 1 predicts disordered region, whilst a score of 0 predicts an ordered region. (D) 1H NMR spectrum of Vps1 Insert B. The signals at 0–0.5 ppm and above 8.5 ppm are characteristic of folded proteins. The large signals at 7.8 and 8.2 ppm come from residual imidazole, and the signal at 5 ppm from water. The sharp signal at 0 ppm is the chemical shift reference compound DSS (4,4-dimethyl -4-silapentane-1-sulfonic acid).

The Insert B region of Vps1 lies in the same position as the PH domain in classical dynamins, between the middle domain and the C-terminal GTPase Effector Domain (GED) region (Fig 1A). Bioinformatic analysis of Insert B in Vps1 and other fungal Vps1 homologues, reveals only a relatively low level of sequence identity, with the basidiomycete *Ustilago maydis* putative Vps1 homologue showing 47% homology over only two thirds of the 76 residues constituting the Vps1 Insert B. The related dynamin-like protein Dnm1 from *S*. *cerevisiae*, which plays a role in mitochondrial fission, only shows homology over the latter half of the Insert B sequence with 24% identity over 50 residues. This level of variation might suggest that different Insert B domains may confer distinct functions or binding specificities to the protein orthologues.

Alignment of more closely related fungal species (Fig 1B) did however reveal identifiable regions of conservation. The alignment suggests two possible subdomains, one at each end of insert B with a less well-conserved region between them. In the N-terminal part of Insert B there is a region of 7 amino acids that is 100% conserved, while the C-terminal region contains a 27 residue stretch with 52% identity (70% similarity) between species. Interestingly, one of the most conserved motifs in the C-terminal region is a run of basic residues, mostly lysines. Such stretches have been observed to be involved in lipid binding in other dynamin family proteins [17, 18].

In terms of structure, the majority of secondary structure prediction software programs predict disorder for Insert B. However, analysis using PONDR (prediction of Natural Disordered regions software) suggests that there is a region of order within its C-terminal half (Fig 1C). We therefore expressed and purified Insert B from *E*.*coli* and subjected it to 1-dimensional NMR. This NMR spectrum also indicates that the domain has characteristics of folded structures (Fig 1D).

### Mutation of Vps1 insert B lysines reduces binding to liposomes

**Fig 2:**
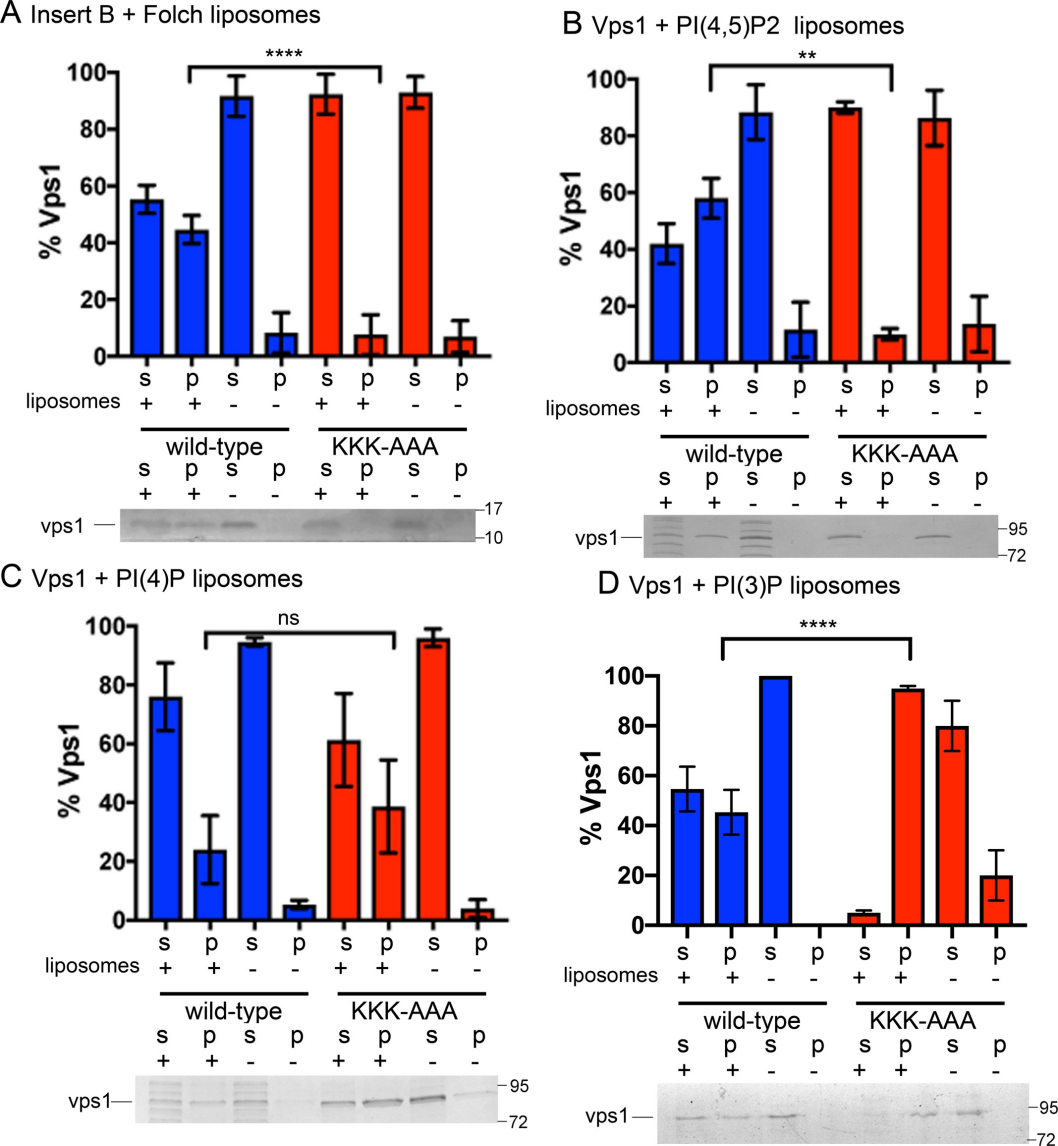
Binding of full length Vps1 and Insert B to liposomes *in vitro*. Insert B from Wild type Vps1 and mutant Vps1 KKK-AAA, and full length Vps1 wild type or KKK-AAA mutant were purified as described in Materials and Methods and incubated in the presence or absence of liposomes. Samples were centrifuged then analysed by SDS–PAGE as supernatant and pellet fractions. (A) Insert B wild type and mutant with total brain extract Folch fraction 1. Only wild type Vps1 Insert B is found in the pellet fraction indicating that the KKK motif is necessary for Insert B binding to lipid. Full length wild type and mutant Vps1 binding and sedimentation with liposomes containing either PI(4,5)P_2_ (B), PI(4)P (C) and PI(3)P (D) as the sole inositol phosphate lipid was then tested. Liposome binding was performed in three independent experiments and errors shown are standard deviation. Each supernatant + pellet band was considered to combine to be 100% of protein used in each experiment. Statistical tests are one-way ANOVAs with Tukey’s post hoc test for multiple comparisons. For gels s—supernatant; p–pellet. +/- indicate presence of liposomes. Full gels are shown in S1 Fig.

Given that stretches of lysines are conserved between different fungi in the C-terminal half of Vps1 Insert B regions (Fig 1B), and the previously reported link between lysine residues and membrane binding, the ability of Insert B to interact with lipids was tested [17, 18]. Expressed and purified Insert B was incubated with liposomes generated from Folch fraction lipids. As shown in Fig 2A, Insert B alone shows clear binding to lipids in this lipid co-sedimentation approach. We next investigated the importance of the conserved lysines for Vps1 binding to membranes. Mutations were generated in both the Insert B alone and in full length Vps1 sequence to mutagenise KKK591-593 to alanines (hereafter called the Vps1 KKK-AAA mutant). Mutations in the Insert B domain alone resulted in an almost complete loss of binding to liposomes (Fig 2A).

To gain insight into the binding capacity and specificity for lipids due to the Insert B lysine stretch, full length Vps1 was also mutagenized in a similar way and both full length wild type and mutant Vps1 were tested for binding to synthetic liposomes made up of PC, PE, and either 20% PI(4,5)P_2_, PI(4)P, or PI(3)P as described in Materials and Methods. As shown in Fig 2B, 2C and 2D, full length wild type Vps1 shows clear binding to liposomes containing PI(4,5)P_2_ and weaker, but reproducible binding to PI(3)P and PI(4)P-containing liposomes. Interestingly, the KKK-AAA Vps1 mutation inhibited the association with the PI(4,5)P_2_-containing liposomes but did not show any significant change in binding to the PI(4)P-containing liposomes while binding to PI(3)P liposomes showed some increase. These data support the idea that the lysine residues within Insert B make a significant contribution to the binding of Vps1 to PI(4,5)P_2_ containing membranes.

### In vivo effect of the Vps1 KKK-AAA mutation on its cell localization

**Fig 3:**
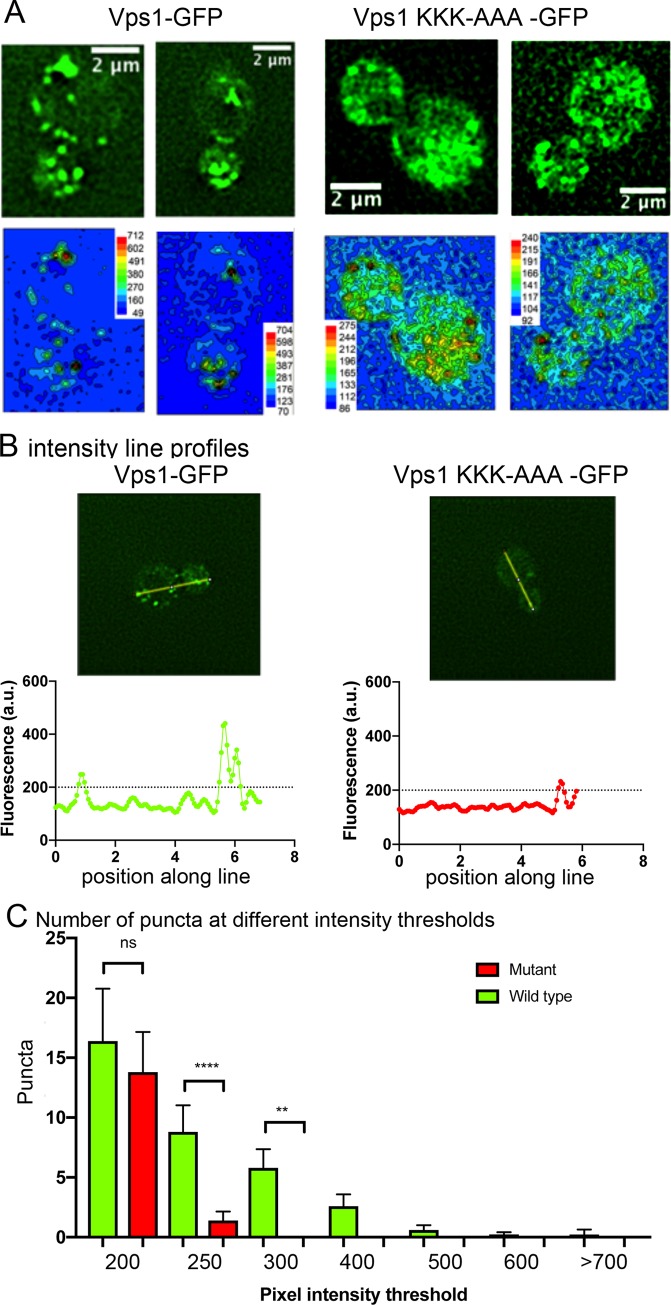
Effect of Vps1 KKK mutation on localization in vivo. (A) GFP-tagged wild type and mutant Vps1 were expressed in a *Δvps1* strain and localization observed as described in the Methods. The mutant protein showed some redistribution to the cytosol though puncta were still visible. Intensity heat maps were generated using the DeltaVision SoftWoRx application (lower panels). These indicate a shift from high intensity puncta for wild type Vps1 to lower intensity puncta in the mutant. (B) Line profiles were drawn through cells as indicated in representative images. The pixel intensity of individual puncta in cells was measured and the number of puncta in each intensity group counted. The combined data is shown in (C). Statistical analysis was used to determine whether the difference between the number of spots in the groups is significantly different between wild type and mutant cells.

While Vps1 binding to liposomes has been previously observed, the importance of lipid binding for localization and recruitment at different cell sites has not been reported. The predominant localization observed for Vps1 is at endosomes while less intense puncta can also be observed at the cell periphery. Thus, we next considered whether the lysine mutations affected localization to any, or all of, these sites. Cells expressing either wild type Vps1 or the Vps1 KKK-AAA mutant fused to GFP as the only source of Vps1, were analysed for localization. As shown in Fig 3, the mutant protein appears more diffuse in the cell cytosol. When analysed using an intensity heatmap, distinct foci of intensity are apparent for wild type Vps1 cells but although some foci of intensity can be distinguished for the mutant, the level of signal intensity from these spots is lower than for foci in cells expressing wild type Vps1. Time lapse movies of Vps1-GFP demonstrate that the puncta in both wild type and mutant are dynamic and can assemble and disassemble at different sites in the cell (S1 Video). The puncta in the mutant cells are however less distinct and it is challenging to observe clear points of assembly and disassembly at any of the sites. To quantify overall changes of Vps1 in cells, line profiles were drawn through cells and pixel intensities measured (Fig 3B). The number of puncta in multiple cells above a background level were counted (marked in Fig 3B). As shown in Fig 3C. while there are a similar level of low intensity puncta that can be discerned, cells expressing mutant Vps1 had significantly fewer puncta with a high intensity of pixel intensity.

**Fig 4:**
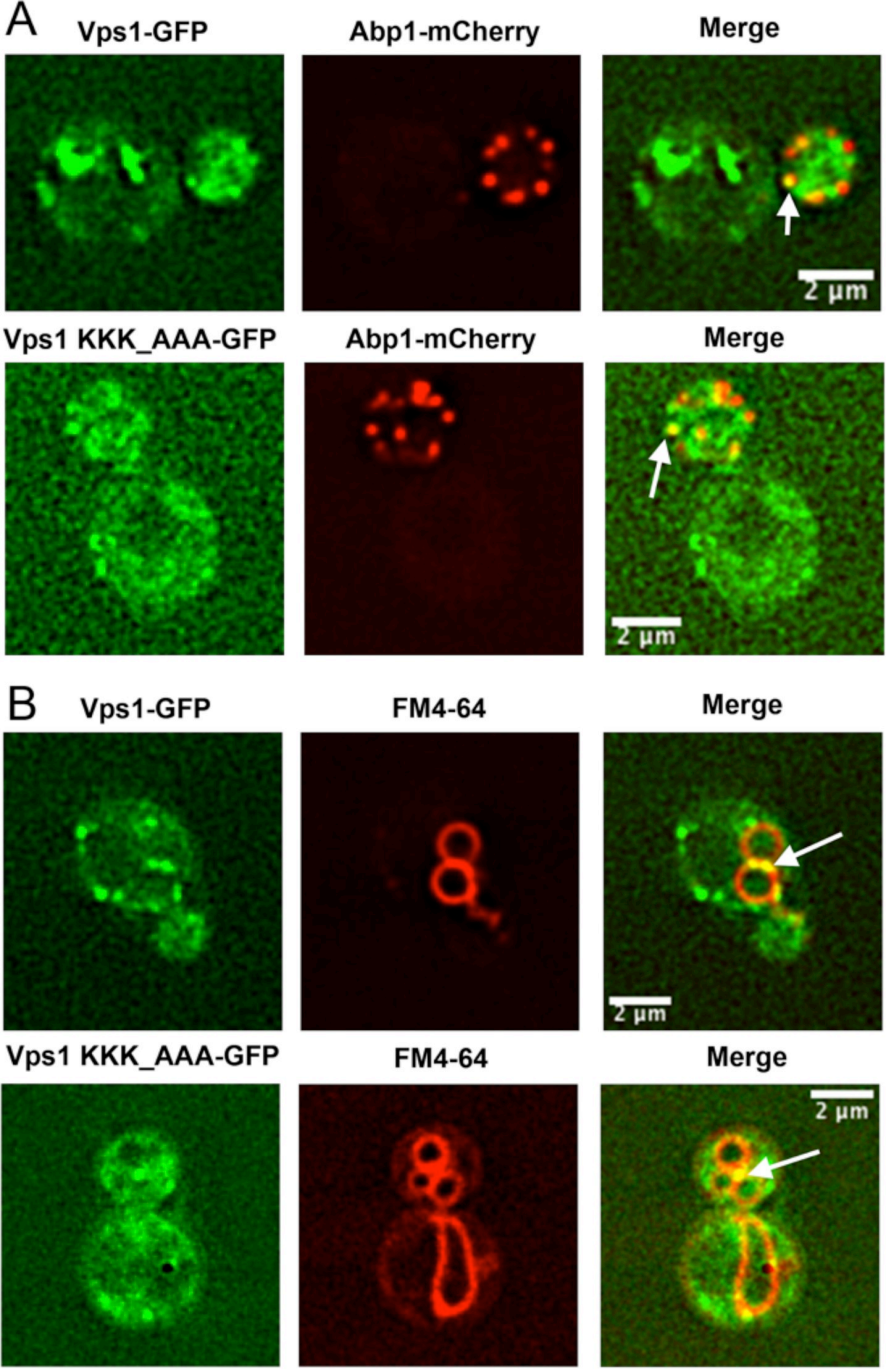
Vps1 co-localisation in cells. Vps1 is known to localize and function at endocytic sites and in late endosomes. (A) To determine whether the KKK mutation affected localization to endocytic sites, GFP-tagged wild type and mutant Vps1 were co-expressed from plasmids in cells otherwise lacking *vps1* with the endocytic marker Abp1-mCherry (KAY1467). As denoted by arrows, peripheral puncta co-staining with Abp1 and Vps1 can be seen for both wild type and mutant protein. (B) FM4-64 is taken up by cells and labels endosomal and vacuolar membranes. Large Vps1-GFP puncta can be observed near the periphery of vacuoles (arrows) in cells expressing either wild type or mutant Vps1.

PI(4,5)P_2_ is considered to be a key lipid required for interacting with proteins during the invagination and scission stages of endocytosis (reviewed in [30]). We analysed cells co-expressing Vps1-GFP and an endocytic marker Abp1-mCherry to determine whether the mutation specifically prevented association with endocytic sites. As shown in Fig 4A localization of Abp1-mCherry and wild type Vps1-GFP is observed. Co-localized spots are also seen for the Vps1 KKK-AAA mutant though these are harder to discern due to the diffuse cytosolic signal. This suggests that while the mutation reduces the ability to bind to PI(4,5)P_2_ in liposome co-sedimentation assays, it does not completely inhibit the ability of Vps1 to become associated with endocytic sites *in vivo* potentially due to the multiple interactions that Vps1 has with endocytic proteins.

Vps1 is also associated with functions including late endosomal fusion and fission events [29, 31–33]. To determine whether the lysine mutations affect localization to late endosomal organelles, cells expressing Vps1-GFP were incubated with the lipophylic dye FM4-64. As shown in Fig 4B the wild type protein localizes to large puncta, some of which are adjacent to the vacuolar membrane. Analysis of cells expressing Vps1 KKK-AAA demonstrates that the vacuoles themselves appear similar to those in wild type cells indicating that the mutation does not cause a defect in Vps1 function with regard to vacuolar fission and fusion function, in contrast to the altered vacuoles seen in *vps1*Δ cells. In addition, the mutant protein is still localized in clear puncta associated with the vacuolar membrane.

### Vps1 lipid binding mutation does not compromise the majority of Vps1 functions

**Fig 5:**
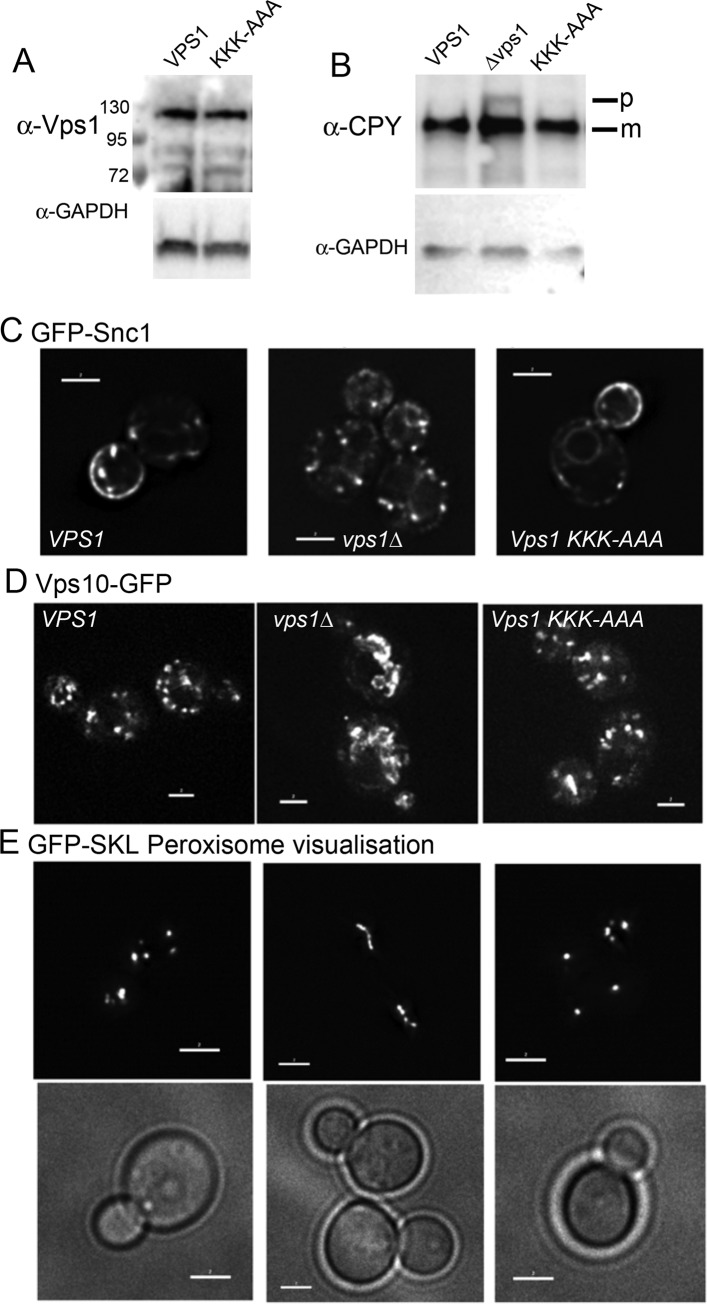
The effect of the Vps1 KKK-AAA mutant on Vps1 function. (A) Expression of wild type Vps1GFP and Vps1(KKK-AAA)-GFP mutant in cells. Western blot showing similar expression of wild type and mutant Vps1. GAPDH levels were used as a control for loading. (B) Processing of carboxypeptidase Y (CPY) requires trafficking to the vacuole. In the absence of Vps1 processing is defective and western blotting for the CPY protein demonstrates an accumulation of partially processed CPY protein (P). This accumulation is not seen in cells expressing wild type or the mutant Vps1 where only the mature (m) form is detected. (C) Localisation of GFP-Snc1-SUC2 fusion was analysed in a *vps1* deletion strain and in the presence wild type Vps1 and Vps1 KKK-AAA mutant to investigate endocytic recycling of the SNARE protein. Scale bars 2 μM. (D) A Vps10-2x GFP *Δvps1* strain was crossed with *Δvps1* strain and strains expressing the wild type or Vps1 mutant to determine whether the mutant is able to rescue the retrograde trafficking phenotype. Scale bars 2 μM. (E) Peroxisomes labelled with GFP peroxisome reporter (GFP-SKL) were visualised in a *Δvps1 Δdnm1* strain expressing plasmids with wild type or mutant versions of Vps1. Images are compressed Z-stacks for fission-proficient strains and in a single plane for fission-deficient strains. Scale bars 2 μM.

In order to determine whether lipid binding facilitated by the Insert B lysines, plays an important role in known Vps1 functions, a Vps1 KKK-AAA mutant was generated and expressed in cells lacking endogenous Vps1. As shown in Fig 5A expression levels were similar to those of cells expressing the wild type protein indicating that the mutation does not induce instability in the protein.

As mentioned above, Vps1 function is required for a number of intracellular membrane remodeling events including trafficking of the vacuolar protein carboxypeptidase Y (CPY) from the Golgi to vacuoles [21]. Deletion of *vps1* also causes a class F vacuolar phenotype with a large vacuole surrounded by multiple small or fragmented vacuolar structures [20–23, 26, 32]. To determine whether the mutation impacted on the functions of Vps1 required for these roles, cells expressing wild type Vps1, the Vps1 KKK-AAA mutant or from which Vps1 had been deleted were analysed for CPY trafficking. If CPY is able to reach the vacuole, the CPY enzyme is processed by cleavage of precursor forms and a mature form is generated. If however CPY is not effectively trafficked to the vacuole, as in the Vps1 deletion strain, a precursor form can be visualized on a blot (Fig 5B). As shown, mutant Vps1 is able to rescue the trafficking defect and only mature CPY is seen on the blots.

The effect of the Vps1 KKK-AAA mutant on endosomal trafficking was also investigated. Previously, we have shown that deletion of Vps1 disrupts normal trafficking of a reporter construct containing the SNARE protein Snc1 fused to both GFP at its N-terminus and *SUC2* conferring invertase activity at its C-terminus [20]. In wild type cells the reporter is found predominantly at the plasma membrane whilst in the *vps1Δ* cells its trafficking is affected and GFP-Snc1 localizes mainly to internal punctate sites (Fig 5C). The Vps1 KKK-AAA mutant rescued the localization of the reporter indicating these residues are not required for trafficking of Snc1.

A role for Vps1 in fission of retrograde carriers from endosomes has also been reported. In the *vps1* deletion strain a Vps10-2xGFP fusion protein undergoes a prominent shift from Golgi and endosomal localization to the vacuolar membrane, similar to the phenotype observed for retromer deficient cells [32]. As shown (Fig 5D), expression of wild type Vps1 and the Vps1 KKK-AAA mutant rescues this phenotype indicating that the lipid binding contribution of these residues is not important for the functionality of this fission event.

Finally, Vps1 has also been demonstrated to function in fission of peroxisomes [26]. These organelles grow and divide by a fission mechanism to ensure appropriate inheritance. In wild type cells, there are usually multiple small peroxisomes distributed throughout the cytoplasm. In the absence of both dynamin-like proteins Vps1 and Dnm1 the number of peroxisomes is greatly reduced, and cells frequently contain a single elongated peroxisome (Fig 5E). This phenotype can be completely rescued by re-expression of Vps1. As shown the mutant Vps1 KKK-AAA also rescued the fission defect of peroxisomes.

### Vps1 lysine mutations compromise endocytic invagination and fission

**Fig 6:**
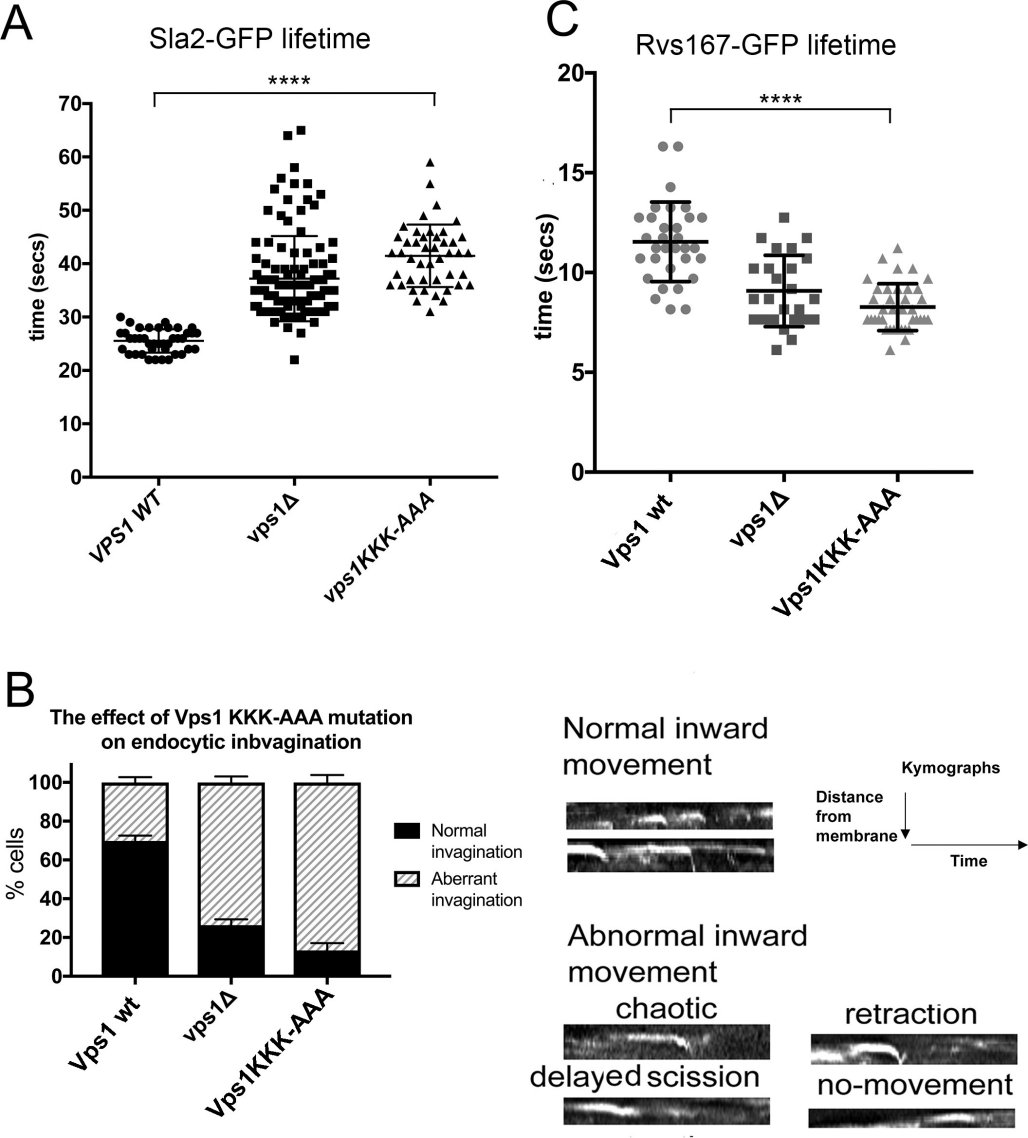
Effect of Vps1 KKK-AAA mutant on endocytosis. (A) Lifetime of Sla2-GFP in the *Δvps1* strain and in the strains expressing wild type or Vps1KKK-AAA mutant. Error bars indicate standard deviation. Statistical analysis using one way ANOVA with Tukeys post-hoc multiple comparison test in GraphPad Prism, indicates life-times in mutant cells are significantly different from those in wild type cells (P value <0.0001) (B) The behaviour of Sla2-GFP in wild type Vps1, and Vps1 mutant cells. Abnormal behaviours included chaotic (with multiple behaviours), retraction, delayed scission, or no invagination. (C) Lifetime of Rvs167-GFP in the *Δvps1* strain and in the strains expressing wild type or Vps1 KKK mutant. Error bars indicate standard deviation. Statistical analysis as above indicates life-times in mutants are significantly different from those in wild type cells (p value <0.0001).

Previous work from our lab and others has demonstrated a role for Vps1 in endocytic fission, potentially working in concert with the BAR domain protein Rvs167 [20, 34]. To determine whether the triple lysine mutation has any effect on endocytosis, the lifetimes of Sla2-GFP puncta at the plasma membrane were measured in the Vps1 deletion strain and in the strains expressing either wild type or the Vps1 KKK-AAA mutant. As shown (Fig 6A) the Vps1 KKK-AAA mutant has a significant increase in the lifetime of Sla2 puncta at the membrane compared to wild type cells (wild type 25.5 s ± 2.2; Vps1 KKK-AAA 41.5 s ± 5.9). Closer analysis of the invagination behaviour of these puncta demonstrated that there was an emergence of aberrant scission behaviours, in particular delayed scission and retraction events such that fewer than 15% of puncta showed normal invagination (Fig 6B).

The yeast BAR domain amphiphysin protein Rvs167, interacts with Vps1 and both proteins contribute to scission of endocytic vesicles ^34^. In a previous study we also demonstrated interactions between Rvs167 and Vps1 that are affected by phosphorylation of Vps1 within the Insert B region, at serine 599 close to the lysine stretch [35]. Mutation of the Vps1-Rvs167 interaction site correlated with a shorter lifetime of Rvs167 association at endocytic sites. Given that the Vps1 KKK-AAA mutation reduces the interaction with PI(4,5)P_2_-containing liposomes, if the interaction is important in vivo we would predict that this reduced membrane interaction could also lead to a reduced lifetime for Rvs167. Thus, we measured lifetimes of Rvs167 at endocytic sites. As shown in Fig 6C the mutation of the lysines correlates with a significantly reduced lifetime for the amphiphysin at the membrane.

## Discussion

The classical dynamins contain a PH domain that has been analysed in detail and has been shown to bind directly to lipids. In the case of dynamin-1 this domain shows a higher affinity of binding for the bisphosphate lipid PI(4,5)P_2_ compared to other phophoinositol lipid headgroups, and this binding is enhanced when dynamin is at least in a dimer (or higher order) organization [16]. During endocytosis, lipid binding is considered critical for dynamin function in mediating the scission of vesicles from the plasma membrane. Other classical dynamins such as Dynamin-2 also have a PH domain that can bind to PI(4,5)P_2_. However, increasing evidence of Dynamin-2 functioning at other membrane trafficking sites that are not as enriched in this lipid has raised questions as to the exact function of lipid binding and how it fits with the overall function of the protein in driving membrane fission reactions [36].

Dynamin-related proteins functioning in mitochondrial fission are also known to interact with membranes and in some cases lipid binding has been associated with lysine residues in the Insert B regions of the protein. These Insert B regions within the greater dynamin family are however highly variable and it is not clear whether lipid binding is a general property of these proteins and what role lipid binding plays.

In this study we have investigated the yeast dynamin Vps1 that functions at several stages of membrane trafficking including endocytosis and in peroxisome fission. We reveal that the Vps1 Insert B region is able to bind to liposomes but that mutation of three sequential lysine residues reduced binding of full length Vps1 to PI(4,5)P_2_ containing liposomes but does not preclude binding to PI(3)P and PI(4)P-containing liposomes indicating further lipid binding regions within the protein.

Analysis of the Vps1 KKK-AAA mutant protein *in vivo* demonstrates that the lysine patch is important for normal levels of membrane association and that when mutated the protein is observed to be more diffuse in the cytosol. Co-localization studies however, indicate that the mutant does retain some ability to associate at both endocytic and later endosomal membrane sites. Given that the mutant protein, when expressed as the sole source of Vps1 in cells, can rescue the majority of functions previously shown to be associated with Vps1, this suggests that the lipid binding interaction identified is not required for these roles. The only function that showed defects with the Vps1 KKK-AAA mutant was endocytosis. In this case the lifetime of an endocytic reporter Sla2-GFP was significantly increased above the lifetime observed in the wild type strain. Given the aberrant kymographic profiles of the Sla2 reporter it appears that there are severe defects with scission in the strain. In this study, we also observed a reduced lifetime of Rvs167 at endocytic sites in the presence of the Vps1 KKK-AAA mutant. Together with other data, our data fit a model in which Vps1 is recruited to endocytic sites through interactions with proteins such as Sla1and possibly with lipids including PI(4)P [23]. As PI(4)P is phosphorylated to PI(4,5)P_2_ by the Mss4 kinase at the plasma membrane Vps1 could encircle and stabilize the forming invagination through an interaction with PI(4,5)P_2_. It has been suggested that the lipid phase boundary forces within endocytic invaginations could contribute to vesicle scission in yeast [37, 38]. Binding of Insert B of Vps1 to PI(4,5)P_2_, along with binding of distinct regions of Vps1 to other lipids such as PI(4)P could result in it utilising this mechanically induced phase boundary to ensure correct micro-positioning of Vps1 at the scission site. The amphiphysin Rvs167 can also bind to PI(4,5)P_2_, as well as to other endocytic proteins and the interaction of Vps1 and Rvs167 also contributes to vesicle scission [34]. We consider it most likely that the reduced association of Vps1 Insert B with PI(4,5)P_2_ at this site may then lead to less stable association of Vps1 to the plasma membrane during invagination, and in turn this reduces the potential for a productive Rvs167 interaction thereby reducing efficiency of scission.

Given that Vps1 functions are associated with lipid fusion/fission it is surprising that the reduction in lipid binding does not cause stronger defects in many pathways and suggests that there are other proteins that are likely to be functioning redundantly in fission. It is noteworthy however, that the ability to bind to PI(3)P and PI(4)P enriched liposomes did not appear to be affected in the full length KKK-AAA mutant. This indicates that the full length Vps1 has the ability to interact with lipid in regions additional to Insert B, and therefore it might only be in its endocytic role that the interaction with PI(4,5)P_2_ via its Insert B region is important. The recent publication of the Vps1 structural architecture reveals some key differences from the classical dynamins but it was proposed that Insert B most likely occupies the same structural space as the PH domain in dynamin, being localised in the central region of the ring structure and in a position to directly contact and encircle the endocytic invagination [39]. Thus, while other Vps1 functions are able to continue or occur redundantly in the presence of a mutagenized Insert B, endocytosis provides a window to investigate the conserved role of a lipid binding motif structurally localized at the centre of the Vps1 oligomeric ring.

## Materials and methods

### Materials

Unless otherwise stated, chemicals were obtained from Sigma-Aldrich (St. Louis, MO). Yeast growth medium (yeast extract, peptone, and agar) was from Melford Laboratories, (Ipswich, Suffolk, United Kingdom), or Sigma (minimal synthetic medium and amino acids). FM4-64 was from Molecular Probes.

### Bioinformatics methods

Regions of natural disorder in Vps1 insert B were predicted using PONDR predictor of natural disordered regions (http://www.pondr.com/).

#### Vps1 purification

Vps1 and Insert B were cloned into pCRT7/Nt-TOPO and were expressed in C43 cells (Lucigen Overexpress C43(DE3) SOLOs) by induction for 7hrs at 37°C with 1mM IPTG. Plasmids used in this study are listed in Table 1. Pelleted cells from 2 L of culture were resuspended in 30 mM Imidazole, 1 x phosphate buffer, and the protein was purified using HisTrap HP Nickel columns (GE Healthcare) following the manufacturer's instructions. Vps1 was then concentrated and buffer exchanged into F-buffer (2 mM Tris, 0.2 mM CaCl2, 10 mM Imidazole, 1 mM EGTA, 2 mM EDTA, 150 mM KCl pH7.2) in a centrifugal filter device (Centriprep, Amicon, Milipore). Approximately 2 mg of protein was obtained from 2 L of cell culture.

**Table 1:** Plasmids used in this study.

Plasmid	Description	Origin/Reference
pKA544	*URA3*, *CEN* with *PGKterm*	KA lab [40]
pKA677	pKA544 with *VPS1* (inc 320bp 5’)	KA lab [40]
pKA1203	pKA544 with *vps1 KKK591-593AAA*	This study
pKA1070	*pVPS1-VPS1-GFP URA3 CEN*	KA lab [40]
pKA1122	*pVPS1-VPS1 KKK591-593AAA–GFP URA3 CEN*	This study
pKA910	*GFP-SKL LEU2 CEN*	KA lab [40]
pKA850	His-tagged Vps1 wild type	KA lab [40]
pKA1202	pKA850 with *vps1 KKK591-593AAA*	This study
pKA973	His-tagged Vps1_535-612_ (Insert B) wild type	This study
pKA1132	pKA973 with *vps1*_*535-612*_ *KKK591-593AAA*	This study

#### ^1^H NMR

Insert B from Vps1 was purified as described and buffer exchanged into 20 mM NaH_2_PO_4_ pH 7.4 before use. 50 μM Vps1 ^1^H 1d NMR was run on a Bruker Avance I 800 MHz NMR spectrometer at 298K. The spectrum was acquired over 24 kHz spectral width with 4K transients. Water suppression was achieved by presaturation during the recycle delay of 1.5 seconds.

#### Preparation of liposomes

22 μl of a 25 mg/ml solution of Folch fraction-1 (Sigma) or a purpose made mixture of 65% PC, 15% PE, and either 20% PI(4)P, PI(3)P or PI(4,5)P_2_ (Avanti Polar Lipids dissolved in chloroform) was prepared, and dried under a nitrogen stream. Liposomes were formed by resuspension of the dried lipids in 200 μl F-buffer (0.2 mM CaCl2, 12 mM Tris/HCl, pH 8.0, 1 mM NaN3, 50 mM KCl, 1 mM MgCl2, 1 mM EGTA) at 60°C for 30 mins with regular agitation.

#### Liposome cosedimentation assays

Purified Vps1 was pre-spun at 350,000*g* for 15 mins (Beckman Ultra centrifuge, TL100 rotor). Pre-spun protein was immediately added to 0.22 mg/ml liposomes to a concentration of 0.4 μM, in F-buffer, (final volume 50 μl). For full length Vps1 the protein-liposome mixture was immediately re-spun at 110,000*g* for 15 mins. For Insert B the protein-liposome mixture was incubated for 30 mins at room temperature, before pelleting the liposomes at 280,000*g* for 15 mins.

After centrifugation supernatants and pellets were separated, and pellets resuspended in 50 μl of F-buffer. Strataclean resin (Stratagene) was then used to concentrate the protein in each sample. 10 μl of Strataclean resin was added to, and briefly mixed with, each sample, before being pelleted by spinning at 8000*g* in a bench top micro-centrifuge for 2 mins. The supernatant was removed and the pellet resuspended in 10 μl of SDS-PAGE loading buffer. Protein was visualised by SDS-PAGE and Coomassie staining. Data was analysed by densitometry using ImageLab software (BioRad).

#### Yeast strains, plasmids and cell growth

Yeast strains used in this study are listed in Table 2. Cells were grown with rotary shaking at 30°C in liquid YPD medium (1% yeast extract, 2% Bacto-peptone, 2% glucose supplemented with 40 μg/ml adenine) or in synthetic medium (0.67% yeast nitrogen base, 2% glucose) with appropriate supplements. Plasmids used in this study are listed in Table 1. KKK591-593AAA point mutations in the *vps1* gene were generated using a site directed mutagenesis kit (Agilent) with plasmids pKA677, pKA1070, pKA850, and pKA973 as the templates. The constructs were then verified by sequencing. Transformations were performed using lithium acetate as described [41]. All strains carrying tags have growth properties similar to control strains. Vps1 protein was detected on western blots of whole-cell extracts using anti-Vps1 antibody (1:2000 dilution) or anti-GFP antibody (anti-mouse, Roche at 1:1000) with GAPDH as a loading control (anti-GAPDH, mouse Invitrogen at 1:1000). Uptake of FM4-64 and Carboxypeptidase Y processing were analyzed as described previously [34]. Pre-cleaned CPY antibodies (Chemicon International) were used at 1:100 dilution.

**Table 2:** Yeast strains used in this study.

Strain number	Genotype	Note
KAY1095	*MATα his3Δ1*, *leu2Δ0*, *lys2Δ*, *ura3Δ0*, *Δvps1*::*KanMx*	KA lab
KAY1096	*MATα his3Δ1*, *leu2Δ0 lys2Δ*, *ura3Δ0*, *dnm1Δ*::*KanMX*, *vps1Δ*::*HIS5*	KA lab
KAY1337	*MATa RVS167-GFP*::*HIS3*, *his3Δ1*, *leu2Δ0*, *met15Δ0*, *ura3Δ0*, *vps1*::*LEU2*	KA lab
KAY1459	*MATa SLA2-GFP*::*HIS3*, *his3Δ1*, *leu2Δ0*, *met15Δ0*, *ura3Δ0*, *vps1*::*LEU2*	KA lab
KAY1467	*MATα ABP1- mCherry*::*HIS*, *his3Δ1*, *leu2Δ0*, *lys2Δ*, *ura3Δ0*, *vps1Δ*::*KanMx*	KA lab
KAY1463	*MATα his3Δ1*, *leu2Δ0*, *lys2Δ*, *ura3Δ0*, *Δvps1*::*KanMx*, *GFP-Snc1-SUC2 URA3*	KA lab
KAY1894	*MATa/α ura3-52/ura3Δ0*, *leu2-3*,*112/leu2Δ0*, *his3Δ200/his3Δ1*, *lys2-801/lys2Δ*, *trp1-1/TRP1*, *vps1Δ*::*KanMX/VPS1*, *VPS10-2 × GFP*	KA lab [35]
KAY1895	*MATa/*α *ura3-52/ura3Δ0*, *leu2-3*,*112/leu2Δ0*, *his3Δ200/his3Δ1*, *lys2-801/lys2Δ*, *trp1-1/TRP1*, *vps1Δ*::*KanMX/vps1Δ*::*URA3*, *VPS10-2 × GFP*	KA lab [35]
KAY1960	*MATa/*α *ura3-52/ura3Δ0*, *leu2-3*,*112/leu2Δ0*, *his3Δ200/his3Δ1*, *lys2-801/lys2Δ*, *trp1-1/TRP1*, *vps1Δ*::*KanMX/vps1 KKK591-593AAA*, *VPS10-2 × GFP*	This study

#### Fluorescence microscopy

For co-localisation and live-cell imaging, cells expressing tagged proteins were visualized after growing to early log phase in synthetic medium with appropriate supplements.

Epifluorescence microscopy was performed using Olympus IX-81 inverted microscope with a DeltaVision RT Restoration Microscopy System (using a 100x/1.40 NA oil objective), Photometrics Coolsnap HQ camera with Imaging and Image capture performed using SoftWoRx image analysis and model-building application (Applied Precision Instruments, Seattle). Time-lapse live cell imaging of GFP-tagged Sla2 was performed with 1 sec time-lapse. All image data sets were deconvolved, using the SoftWoRx application.

Time-lapse live cell images of Rvs167-GFP was acquired using OMX DeltaVision V4 and a 60xUSPLAPO (numerical aperture, 1.42) objective with refractive index 0.1514 immersion oil (Cargille). Samples were illuminated using Insight Solid State Illuminator (10%), and images were taken simultaneously on separate scientific complementary metal oxide semiconductor (sCMOS) cameras (70 ms exposure). Seven 250 nm sections were acquired every 500 ms (181 time points). The stacks were then deconvolved and processed, using SoftWorx, to produce a movie composed of maximum intensity projections at each time point. The Rvs167-GFP lifetime was analyzed from those projections. Movies and kymographs were assembled using Fiji software. Images were exported as TIFF files and image size adjusted to 300 d.p.i. and assembled using Adobe Photoshop CS2.

#### Statistical analysis

Statistical tests unless otherwise stated, are one-way ANOVAs with Dunnets test for multiple comparisons performed using GraphPad Prism. Confidence interval set to 95%. P value style: >0.05 (ns), 0.022 (*), 0.0021 (**), 0.0002 (***), <0.0001 (****).

## Funding information

This work was supported by BBSRC project grants (BB/K002511/1) to KRA. ID NMR was performed by the Biomolecular NMR Facility, University of Sheffield. Imaging work was performed at the Wolfson Light Microscopy Facility, The University of Sheffield, using the OMX and Deltavision microscopes supported by MRC grant MK/K0157531/1. The funding bodies had no role in study design, data collection and interpretation, or the decision to submit the work for publication.

## Supporting information

S1 Fig Liposome binding assays with full length Vps1. These are the full gels for wild type and mutant Vps1 binding to liposomes containing PI(4,5)P2, PI(4)P and PI(3)P. S—supernatant; p–pellet. +/- indicate presence of liposomes
(TIF)

S1 Video Localization and dynamic behavior of wild type and mutant Vps1-GFP. Vps1-GFP and Vps1(KKK-AAA)-GFP were expressed in *vps1* deletion strain and cells imaged with 0.5 sec exposure and 1 sec time lapse. Fiji software was used to assemble the movies with 5 time frames per second. Scale bar: 2μm.
(AVI)
