# Supplementary figures and images for: Mutation of key lysine residues in the Insert B region of the yeast dynamin Vps1 disrupts lipid binding and causes defects in endocytosis
Source: PLoS One. 2019 Apr 22;14(4):e0215102. doi: 10.1371/journal.pone.0215102 (PMC6476499; doi:10.1371/journal.pone.0215102)

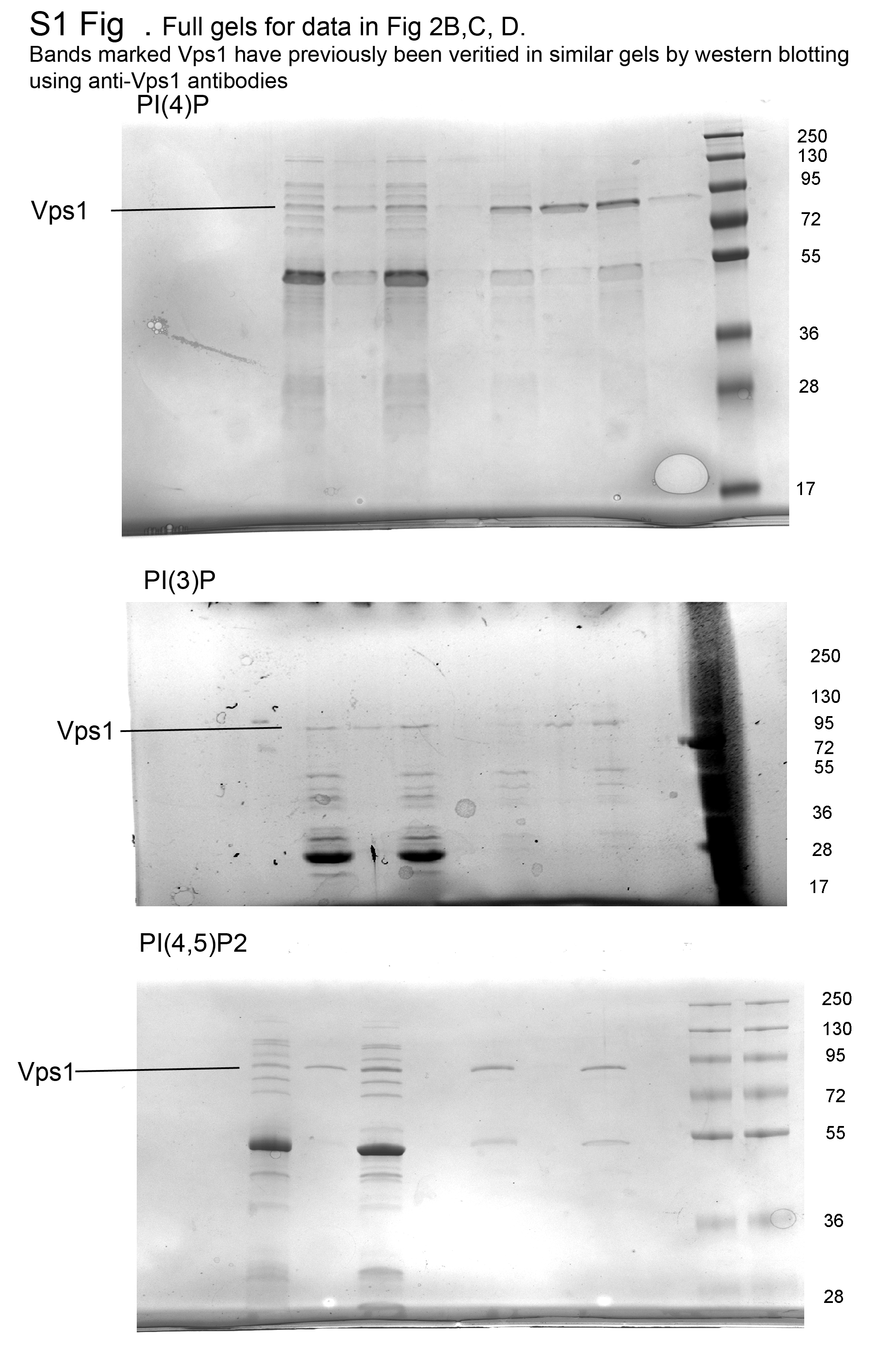

Supplement: S1 Fig — These are the full gels for wild type and mutant Vps1 binding to liposomes containing PI(4,5)P2, PI(4)P and PI(3)P. S—supernatant; p–pellet. +/- indicate presence of liposomes (TIF) [file pone.0215102.s001.tif]
